# Esophageal metal stent for malignant obstruction after prior radiotherapy

**DOI:** 10.1038/s41598-021-81763-x

**Published:** 2021-01-22

**Authors:** Hiroyoshi Iwagami, Ryu Ishihara, Sachiko Yamamoto, Noriko Matsuura, Ayaka Shoji, Katsunori Matsueda, Takahiro Inoue, Muneaki Miyake, Kotaro Waki, Hiromu Fukuda, Yusaku Shimamoto, Mitsuhiro Kono, Hiroko Nakahira, Satoki Shichijo, Akira Maekawa, Takashi Kanesaka, Yoji Takeuchi, Koji Higashino, Noriya Uedo

**Affiliations:** Department of Gastrointestinal Oncology, Osaka International Cancer Institute, 3-1-69 Otemae, Chuo-ku, Osaka, 541-8567 Japan

**Keywords:** Cancer, Gastroenterology

## Abstract

The association between severe adverse events (SAEs) and prior radiotherapy or stent type remains controversial. Patients with esophageal or esophagogastric junctional cancer who underwent stent placement (2005–2019) were enrolled in this retrospective study conducted at a tertiary cancer institute in Japan. The exclusion criteria were follow-up period of < 1 month and insufficient data on stent type or cancer characteristics. We used Mann–Whitney’s U test for quantitative data and Fisher’s exact test for categorical data. Multivariate analysis was performed using a logistic regression model. 107 stents were placed. Low radial-force stents (L group) were used in 51 procedures and high radial-force stents (H group) in 56 procedures. SAEs developed after nine procedures, the median interval from stent placement being 6 days (range, 1–141 days). SAEs occurred more frequently in the H (14%: 8/56) than in the L group (2%: 1/51) (P = 0.03). In patients who had undergone prior radiotherapy, SAEs were more frequent in the H (36%: 4/11) than in the L group (0%: 0/13) (P = 0.03). Re-obstruction and migration occurred after 16 and three procedures, respectively; these rates did not differ significantly between groups (P = 0.59, P = 1, respectively). Low radial-force stents may reduce the risk of SAEs after esophageal stenting.

## Introduction

Esophageal cancer is the eighth most common cancer and the sixth leading cause of cancer-related death worldwide^1^. Esophageal cancer is usually detected at an advanced stage, and only 15–20% of patients undergo successful surgical resection^2^. Thus, palliative management is the best option for patients with unresectable esophageal cancer. Dysphagia caused by obstruction is the predominant symptom, occurring in up to 70% of patients^3^ and prejudicing affected patients’ systemic condition and quality of life.

Radiotherapy (RT), including brachytherapy; chemoradiotherapy (CRT); chemotherapy; and self-expandable metallic stent (SEMS) placement are used to relieve obstruction. Prior studies have assessed the role of these treatments in improving dysphagia^4,5^. RT/CRT may provide long-term relief of dysphagia; however, there is often a long lag time between treatment initiation and symptomatic relief^6^, whereas stent placement provides immediate relief of dysphagia and improves quality of life and is therefore, widely accepted as an alternative treatment. Stent placement can provide rapid and effective palliation of dysphagia^7,8^. However, adverse events associated with stent placement occur at a frequency of 5–65%^8–11^.

Prior RT has been reported as a factor associated with adverse events in many studies^11–13^. The reported stent-related mortality ranges from 0 to 54% in patients treated with CRT prior to SEMS placement compared with 0% to 6% in patients without prior CRT^11,14^. In addition, adverse events associated with stents, such as esophagitis, dehydration, anorexia, migration and fistula formation, occur more frequently in patients who have undergone previous CRT than in those who have undergone stent placement alone^15,16^. Conversely, a meta-analysis^17^ found no relationship between adverse events after stent placement and prior RT or CRT. However, this meta-analysis included many subjects who had undergone chemotherapy alone; thus, the association between adverse events after stent placement and prior RT or CRT remains controversial.

One study reported the advantages and drawbacks of some commonly used SEMS^18^. Previous studies have investigated the association between stent type and adverse events^12,19–23^. Some studies^19–21^ failed to identify a significant association between stent type and adverse events whereas in other studies Gianturco-Z stents as compared with Ultraflex stents and Flamingo Wallstents^22^; Ultraflex stents as compared with Covered Evolution stents^23^; and Ultraflex stents as compared with other types of stents^12^, were associated with a greater number of complications. However, the association between risk of complications and previous RT/CRT was not analyzed in these studies. In this present study, we initially evaluated the risk of adverse events with regard to previous RT/CRT and then compared the risk of adverse events between two types of stents.

## Patients and methods

### Study design and patients

This was a retrospective study conducted at a tertiary cancer institute in Japan. Drawing from the database of patients who had undergone SEMS placement for malignant obstruction of the esophageal or esophagogastric junctional cancer at Osaka International Cancer Institute, we enrolled patients who met the following inclusion and exclusion criteria. The inclusion criterion was esophageal or esophagogastric junctional cancer in patients who had undergone SEMS placement from September 2005 to September 2019. The exclusion criteria were as follows: (1) follow-up period < 1 month; and (2) insufficient data on stent type or cancer characteristics. The food intake of patients was evaluated using the following dysphagia scores (DS)^24^: 0 = able to eat a normal diet; 1 = able to eat some solid foods; 2 = able to eat semi-solid foods; 3 = able to swallow liquids only; and 4 = unable to swallow anything. All study participants provided informed consent. The study protocol was approved by the Institutional Review Board of Osaka International Cancer Institute on 16 January 2020 (No. 19191), and the study was performed in accordance with the Declaration of Helsinki.

### SEMS placement procedure

SEMS placement was conducted under intravenous sedation with midazolam and pethidine hydrochloride or pentazocine. Using a nasal endoscope (GIF-XP260N or GIF-XP290N; Olympus, Japan), we initially checked the oral end of the stenosis and attempted to pass the endoscope through it. If we succeeded in passing the endoscope through the stenosis, we measured the distance between the superior and inferior ends of the stenosis. If we could not pass the endoscope through the stenosis, we measured the stenosis length under fluoroscopy after injection of contrast medium through the endoscopic channel of a catheter for endoscopic retrograde cholangiopancreatography. We then inserted a guide-wire through the endoscopic channel, passed it through the stenosis, and placed its tip in the stomach or duodenum. The superior and inferior margins of the tumor under fluoroscopy were marked using short radio-opaque sticks attached to the patient’s body surface.

### Definitions

In our facility four types of stents (Niti-S [Taewoong Medical, Korea], Evolution [Cook Japan, Japan], Ultraflex [Boston Scientific, Japan), and Hanaro [Boston Scientific]) had been inserted. We classified stents based on their radial force as follows: high radial force group (Ultraflex and Hanaro) and low radial force group (Niti-S and Evolution)^25,26^ (Supplementary Table 1). The low and high radial force groups were divided by the cutoff value of ≥ 38 or ≤ 32 N of radial force at 15 mm expansion in accordance with the recommendations in a previous report^25^. Adverse events included fever, high C-reactive protein concentration (> 10 mg/dL), pain and severe adverse events (SAEs), these including hemorrhage, perforation/mediastinal emphysema, and severe pain. Hemorrhage was defined as hematemesis and/or melena after stent placement, that were considered stent-related complications. Perforation/mediastinal emphysema were defined as perforation occurring after stent placement. In the absence of any symptom or identification of perforation immediately after stent placement, detection of abnormal air or liquid in the para-esophageal space by computed tomography or radiography was considered to denote perforation/mediastinal emphysema. Severe pain was defined as pain that developed within a week of stent placement and required narcotic drugs. Re-obstruction was defined as severe dysphagia that required stent re-placement, total parenteral nutrition, or percutaneous endoscopic gastrostomy. The follow-up period was defined as the period from the day of stent placement to final confirmation that the patient was still alive.

### Statistical analysis

Quantitative data are expressed as median (range) and were compared using Mann–Whitney’s *U* test. Categorical data were compared using Fisher’s exact test. Multivariate analysis was performed using logistic regression and factors that were significant in univariate analysis together with basic factors such as age and sex. A P value of < 0.05 was considered to denote statistically significant. Statistical analyses were performed using EZR (Saitama Medical Center, Jichi Medical University, Saitama, Japan), which is a graphical user interface for R (version 3.3.3; R Foundation for Statistical Computing, Vienna, Austria)^27^.

### Statement of ethics

All study participants provided informed consent. The study protocol was approved by the Institutional Review Board of Osaka International Cancer Institute on January 16, 2020 (No. 19191), and the study was performed in accordance with the Declaration of Helsinki.

## Results

### Characteristics of patients and procedures

**Figure 1:**
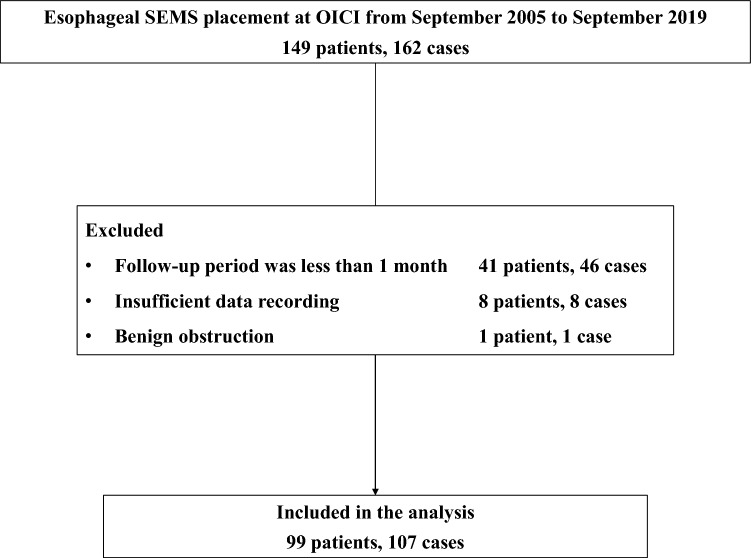
Patients’ enrollment.

During the study period, 149 patients underwent SEMS placement, 99 of whom met the inclusion and exclusion criteria (Fig. 1). We used 18-mm diameter stents in all procedures, fully-covered, partially-covered, and uncovered stents being used in two, 96 and nine of the 107 cases, respectively. We did not compare the stents in terms of coverings because their properties varied. We did not perform bronchoscopy prior to planned stenting. Four patients had undergone two SEMS placements and two had undergone three placements. Thus, 107 SEMS placement procedures were conducted in 99 patients. Ultraflex, Hanaro, Niti-S, and Evolution stents were used in 31, 25, 50, and one case, respectively. Of 107 procedures, 51 procedures in 45 patients were conducted using low radial force stents, while 56 procedures in 54 patients were conducted using high radial force stents.

Table 1 shows patients characteristics and procedures according to study group. There were significant differences in sex, stenosis length, and stent length between the two groups. The high radial force stent group had a higher proportion of men and longer stenosis and stent lengths than the low radial force stent group. Eighty-three procedures were conducted on patients with no history of RT/CRT and the remaining 24 on patients who had undergone RT/CRT for esophageal cancers. We did not perform RT or CRT after SEMS placement.

**Table 1:** Clinical characteristics of patients and pathological characteristics of lesions.

	Low radial force group 51 cases in 45 patients	High radial force group 56 cases in 54 patients	P value
**Age**	68 (32–87)	66 (43–91)	0.686
**Sex**			
Male	31 (68.9)	48 (88.9)	0.022
Female	14 (31.1)	6 (11.1)	
**Tumor location**			
Ce	0	1 (1.9)	0.341
Ut	9 (20.0)	4 (7.4)	
Mt	12 (26.7)	18 (33.3)	
Lt	11 (24.4)	19 (35.2)	
Ae	11 (24.4)	10 (18.5)	
Anastomosis	2 (4.4)	2 (3.7)	
**Macroscopic type**			
Type 1	7 (15.6)	3 (5.6)	0.296
Type 2	11 (24.4)	13 (24.1)	
Type 3	13 (28.9)	23 (42.6)	
Type 4	14 (31.1)	14 (25.9)	
Type 5	0	1 (1.9)	
**Histological type**			
Squamous cell carcinoma	31	43	0.333
Adenocarcinoma	11	10	
Unknown	3	1	
**Secondary cancer**			
Lung cancer	1 (2.2)	0	1
Breast cancer	1 (2.2)	0	
**Prior RT**			
Yes	13 (25.5)	11 (19.6)	0.495
No	38 (74.5)	45 (80.4)	
**Degree of stenosis**^**a**^			
Possible	48 (94.1)	47 (83.9)	0.128
Impossible	3 (5.9)	9 (16.1)	
**Stenosis length, cm**	3 (1–13)	5 (2–15)	0.001
**Stent length, cm**	10 (8–15)	10 (7–15)	0.015
**Follow-up period, month**	3 (1–12.0)	2 (0.5*–12.5)	0.237

Data are presented as median (range) or n (%).

Quantitative data were compared using Mann–Whitney’s U test.

Categorical data were compared using Fisher’s exact test.

Ae, abdominal esophagus; Ce, cervical esophagus; Lt, lower thoracic esophagus; Mt, middle thoracic esophagus; Ut, upper thoracic esophagus.

^a^Stenosis was evaluated by an approximately 6 mm diameter nasal endoscope.

*Adverse events of less than 1 month are included.

### Adverse events, SAE and re-obstruction

Adverse events developed after 34 procedures with a median interval of 6 days (range, 1–141 days) from the day of stent placement. Adverse events tended to develop more frequently in the high radial force (39%, 22/56) than in the low radial force stent group (24%, 12/51); however, the difference was not significant (P = 0.098).

Nine of the 34 adverse events were classified as SAEs, five being hemorrhage, three perforation/mediastinal emphysema, and one severe pain. The incidence of SAEs tended to be higher in patients who had undergone RT/CRT (17%, 4/24 procedures) than in those who had not RT/CRT (6%, 5/83 procedures); however, this difference was not statistically significant (P = 0.11).

When we compared the incidence of SAEs by stent type (Table 2), we found that SAEs developed more frequently in the high radial force (14%, 8/56 procedures) than in the low radial force stent group (2%, 1/51 procedures) (P = 0.03). In the subgroup of patients who had undergone prior RT/CRT, SAEs also developed more frequently in the high radial force (36%: 4/11 procedures) than in the low radial force stent group (0%, 0/13 procedures) (P = 0.03). Multivariate analysis showed an association between high radial force stents and SAEs (Table 3).

**Table 2:** Outcome of the two groups.

	Low radial force group 51 cases in 45 patients	High radial force group 56 cases in 54 patients	P value
**Severe adverse event**			
Yes	1 (2)	8 (14.3)	0.033
No	50 (98)	48 (85.7)	
**Severe adverse event in patients with prior RT/CRT**			
Yes	0	4 (36.4)	0.031
No	13 (100)	7 (63.6)	
**Re-obstruction**			
Yes	9 (17.6)	7 (12.5)	0.589
No	42 (82.4)	49 (87.5)	
**Migration**			
Yes	1 (2)	2 (4)	1
No	50 (98)	54 (96)	

Data are presented as n (%).

These data were compared using Fisher’s exact test.

**Table 3:** Clinical factors associated with SAEs.

	Univariate analysis		Multivariate analysis	
	Odds ratio (95% CI)	P value	Odds ratio (95% CI)	P value
**Age**	–	0.563	0.96 (0.897–1.04)	0.329
**Sex**				
Male/female (reference)	0.95 (0.164–10.090)	1	0.20 (0.023–1.74)	0.145
**Radial force**				
High/low (reference)	8.20 (1.034–376.318)	0.033	21.8 (1.510–314.00)	0.024
**Prior RT/CRT**				
Yes/no (reference)	3.08 (0.558–15.813)	0.112	5.99 (1.130–31.70)	0.035
**Tumor location**				
Ce/Ut/Mt/Lt/Ae/anastomosis	–	0.962	–	–
**Macroscopic type**				
Type 1/2/3/4/5	–	0.591	–	–
**Degree of stenosis**^**a**^				
Possible/impossible(reference)	1.01 (0.115–48.912)	1	–	–
**Stenosis length**	–	0.238	0.975 (0.743–1.28)	0.855

Quantitative data was compared using Mann–Whitney’s U test.

Univariate analysis was compared using Fisher’s exact test.

Multivariate analysis was performed using logistic regression.

Ae, abdominal esophagus; Ce, cervical esophagus; Lt, lower thoracic esophagus; Mt, middle thoracic esophagus; Ut, upper thoracic esophagus.

^a^Stenosis was evaluated by an approximately 6 mm diameter nasal endoscope.

**Figure 2:**
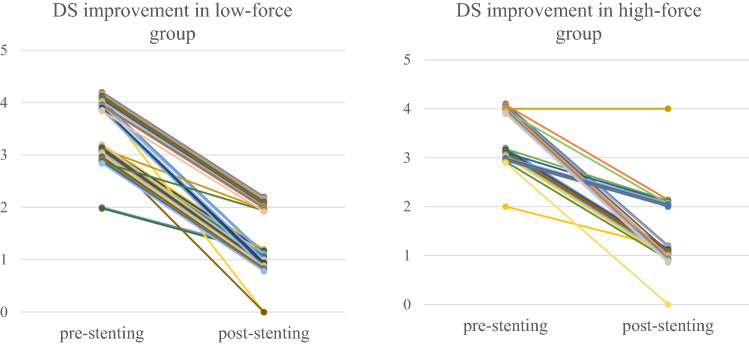
Degree of dysphagia score improvement in two groups.

Re-obstruction and migration occurred after 16 and three procedures, respectively (Table 2). The rates of re-obstruction and migration did not differ significantly between the two groups (P = 0.59 and P = 1, respectively). Dysphagia scores were recorded in 72 patients, including 45 patients in the low radial force and 27 in the high radial force group. The median improvement in dysphagia score was 2 (range, 0–3). There was no significant difference in improvement in dysphagia score between the high radial force and low radial force stent group (P = 0.82) (Fig. 2). The median time to dysphagia recurrence from stent placement was 4 months (range, 1–5.5 months) in the high radial force stent group and 6 months (range, 2–12.0 months) in the low radial force stent group; this difference was not significant (P = 0.08). Multivariate analysis failed to identify any factors associated with re-obstruction (Table 4).

**Table 4:** Clinical factors associated with re-obstruction.

	Univariate analysis		Multivariate analysis	
	Odds ratio (95% CI)	P value	Odds ratio (95% CI)	P value
**Age**	–	0.567	0.98 (0.943–1.03)	0.444
**Sex**				
Male/female (reference)	0.79 (0.208–3.759)	0.744	0.88 (0.232–3.31)	0.846
**Radial force**				
High/low (reference)	0.67 (0.194–2.219)	0.589	0.83 (0.246–2.78)	0.760
**Prior RT/CRT**				
Yes/no (reference)	1.18 (0.250–4.480)	0.753	–	–
**Tumor location**				
Ce/Ut/Mt/Lt/Ae/anastomosis	–	0.907	–	–
**Macroscopic type**				
Type 1/2/3/4/5	–	0.724	–	–
**Degree of stenosis**^**a**^				
Possible/impossible (reference)	0.87 (0.158–8.955)	1	0.82 (0.154–4.32)	0.810
**Stenosis length**	–	0.432	0.90 (0.705–1.14)	0.366

Quantitative data was compared using Mann–Whitney’s U test.

Univariate analysis was compared using Fisher’s exact test.

Multivariate analysis was performed using logistic regression.

Ae, abdominal esophagus; Ce, cervical esophagus; Lt, lower thoracic esophagus; Mt, middle thoracic esophagus; Ut, upper thoracic esophagus.

^a^Stenosis was evaluated by passing an approximately 6 mm diameter nasal endoscope.

We analyzed patients with squamous cell carcinoma and adenocarcinoma of the esophagogastric junction, separately. Table 5 shows the outcomes of patients with squamous cell carcinoma. SAEs developed more frequently in patients in the high radial force (16%, 7/44 procedures) than low radial force stent group (3%, 1/35 procedures); this difference was not significant (P = 0.07). In the subgroup of patients who had undergone prior RT/CRT, SAEs developed more frequently in the high radial force (40%, 4/10 procedures) than low radial force stent group (0%: 0/12 procedures) (P = 0.03). There was no significant difference in re-obstruction and migration between the two groups. Table 6 shows the outcomes of patients with carcinoma of the esophagogastric junction.

**Table 5:** Outcome of patients with squamous cell carcinoma.

	Low radial force group 35 cases in 31 patients	High radial force group 44 cases in 43 patients	P value
**Severe adverse event**			
Yes	1 (3)	7 (16)	0.070
No	34 (97)	37 (84)	
**Severe adverse event in patients with prior RT/CRT**			
Yes	0	4 (40)	0.028
No	12 (100)	6 (60)	
**Re-obstruction**			
Yes	6 (17)	7 (16)	1
No	29 (83)	37 (84)	
**Migration**			
Yes	0	1 (2)	1
No	35 (100)	43 (98)	

Data are presented as n (%).

These data were compared using Fisher’s exact test.

**Table 6:** Outcome of patients with adenocarcinoma of the esophagogastric junction.

	Low radial force group 14 cases in 11 patients	High radial force group 10 cases in 10 patients	P value
**Severe adverse event**			
Yes	0	1 (10)	0.417
No	14 (100)	9 (90)	
**Severe adverse event in patients with prior RT/CRT**			
Yes	0	0	1
No	1 (100)	1 (100)	
**Re-obstruction**			
Yes	3 (21)	0	0.239
No	11 (79)	10 (100)	
**Migration**			
Yes	1 (7)	1 (10)	1
No	13 (93)	9 (90)	

Data are presented as n (%).

These data were compared using Fisher’s exact test.

## Discussion

In this study, we found that the risk of SAEs differed depending on the type of stent used. Many previous studies have investigated the risk of SAEs for various types of stent^12,19–23^. Some of these studies found significant difference, whereas others did not^19–21^. In the studies that did identify significant differences^12,22,23^, higher rates of SAEs were associated with higher radial force stents^28,29^. The results of these studies^12,22^ regarding the relationship between the risk of SAEs and radial force are in agreement with the present findings.

The association between the mechanical properties of esophageal stents and clinical outcome is poorly understood. Stents vary in their material, diameter, radial force, axial force, and the presence or absence of covering^29,30^. Among these factors, diameter and radial force may be the most important determinants of outcomes. Pressure on the esophageal wall is mainly determined by the stent diameter and radial force. Inserted stents initially attach to the esophagus by radial force. The stent then expands to a specified diameter and becomes fixed to the esophagus by development of adjacent fibrosis and granulation. In our study, all stents had the same diameter (18 mm), whereas radial force varied (high radial force versus low radial force). We found no significant differences in improvement in dysphagia score, re-obstruction rate, or migration rate between the high radial force and low radial force stent groups. Given that there were fewer SAEs in the low radial force stent group, and that there were no significant differences in efficacy or risk-related variables (i.e., dysphagia score, re-obstruction rate, and migration rate), we recommend low radial force stents for the relief of symptoms of obstruction caused by esophageal or esophagogastric cancer.

In this study, there was a non-significant tendency for the incidence of SAEs to be higher in patients who had undergone previous RT/CRT (17%, 4/24 procedures) than in those who had not (6%, 5/83 procedures). Esophageal RT/CRT can cause vasculitis, hypoxemia, and fragility of the esophageal wall^31,32^ that can manifest as esophagitis, ulcer, fibrosis, and stricture. Considering that only a few patients (24) had undergone prior RT/CRT, the difference in incidence of SAEs between the two groups may have been significant if the patient cohort had been larger. Thus, clinicians should be aware of the risk of SAEs after stent placement in patients who have undergone prior RT/CRT. Stents with a higher radial force may better stabilize the stent position; however, the strong compression may also cause fragility of the esophageal wall. In support of this contention, the incidence of SAEs was significantly higher in the high radial force (36.4%, 4/11 patients) than the low radial force stent group (0%, 0/13 patients).

The results of our study indicate that low radial force stents may be a better option, especially for patients who have undergone prior RT/CRT. However, a randomized comparing low radial force and high radial force stents is required to determine more definitely whether this is true. In addition, there is a need for more data on the use of low radial force stents in patients who have undergone prior RT/CRT. Regarding additional treatment after stenting, Tinusz et al.^33^ reported that the benefit of additional oncological treatment alongside stenting is unclear; however, they found no association between oncological treatment and the rate of complications. These findings suggest that stenting is a valid treatment option for obstruction caused by esophageal cancer.

One strength of this study is that we found a significant difference between two types of stents, the difference being more pronounced in patients who had undergone prior RT. However, our study had several limitations that should be highlighted. First, this was a retrospective study performed in a single institution. The characteristics of the high radial force and low radial force stent groups differed considerably, which may have caused some bias in the comparison. Second, the total number of patients, especially the number who had undergone prior RT/CRT, was small. Third, the number of patients who were followed up was small. Many of the observed adverse events occurred within 1 month of stent placement. We excluded patients with a follow up period of < 1 month because we thought that a minimum of 1 month follow up would be required to assess complications. Stent placement is a palliative form of treatment of cancer. Follow-up of some patients was difficult because they were transferred to palliative care facilities soon after stent placement. Fourth, the scientific value of classifying stents based on radial force has not been investigated. Further analysis is required to determine whether there is a significant association between radial force and adverse events.

In conclusion, we found that low radial force stents were associated with fewer SAEs and were not associated with worse dysphagia scores, re-obstruction rates, or migration rates.

## Supplementary Information

Supplementary Table S1.

## Data Availability

All data generated or analyzed during this study are included in this published article.
