# Supplementary material for: Esophageal metal stent for malignant obstruction after prior radiotherapy
Source: Sci Rep. 2021 Jan 22;11:2134. doi: 10.1038/s41598-021-81763-x (PMC7822838; doi:10.1038/s41598-021-81763-x)
Supplement: Supplementary file 1 — Supplementary Table S1. [file 41598_2021_81763_MOESM1_ESM.docx]

Esophageal metal stent for malignant obstruction after prior radiotherapy

Hiroyoshi Iwagami^1^, Ryu Ishihara^1*^, Sachiko Yamamoto^1^, Noriko Matsuura^1^, Ayaka Shoji^1^, Katsunori Matsueda^1^, Takahiro Inoue^1^, Muneaki Miyake^1^, Kotaro Waki^1^, Hiromu Fukuda^1^, Yusaku Shimamoto^1^, Mitsuhiro Kono^1^, Hiroko Nakahira^1^, Satoki Shichijo^1^, Akira Maekawa^1^, Takashi Kanesaka^1^, Yoji Takeuchi^1^, Koji Higashino^1^, Noriya Uedo^1^.

^1^Department of Gastrointestinal Oncology, Osaka International Cancer Institute, Osaka, Japan

***Corresponding Author:** Ryu Ishihara, MD, PhD

Department of Gastrointestinal Oncology, Osaka International Cancer Institute

3-1-69 Otemae, Chuo-ku, Osaka 541-8567, Japan.

Tel: +81-6-6945-1181

Fax: +81-6-6945-1902

E-mail: [ryu1486@gmail.com](mailto:ryu1486@gmail.com)

**Supplementary table 1:** Relevant stent variables

|  | newton of radial force at 15mm expansion |
| --- | --- |
| High rafial force stent group |  |
| Ultraflex  Hanaro | 79  38 |
| Low rafial force stent group |  |
| Niti-S  Evolution　partially covered, fullcovered | 8  25, 29 |
